# Valorization of Waste Water of *Rosa damascena* Oil Distillation Process by Ion Exchange Chromatography

**DOI:** 10.1155/2020/5409493

**Published:** 2020-11-30

**Authors:** Zahra Sabahi, Fatemeh Farmani, Elham Mousavinoor, Mahmoodreza Moein

**Affiliations:** ^1^Medicinal Plants Processing Research Center, Shiraz University of Medical Sciences, Shiraz, Iran; ^2^Department of Pharmacognosy, School of Pharmacy, Shiraz University of Medical Sciences, Shiraz, Iran

## Abstract

Water steam distillation is a classical method of rose oil production from the flowers of *Rosa damascena* Mill. This process produces considerable amount of waste water. In this study, ion-exchange column chromatography (Amberlite was the stationary phase) was used to prepare polyphenol-enriched fraction of waste water with improved biological activity. Phenol, flavonoid, and anthocyanin contents were examined before and after using column. Antioxidant activities, DNA protection ability, xanthine oxidase inhibition, and cytotoxicity of this fraction were also determined. The use of Amberlite increased phenol, flavonoid, and anthocyanin contents in fraction compared to the sample before fractionation. The IC_50_ values of various antioxidant assays comprises 2,2-diphenyl-1-picryl-hydrazyl-hydrate (DPPH), ferric-reducing antioxidant power assay (FRAP), and 2,2′-azino-bis(3-ethylbenzothiazoline-6-sulfonic acid) (ABTS) which were 226.66 ± 1.25, 126.03 ± 0.11, and 241.43 ± 0.33 for waste water, and these values for fraction were 63.21 ± 0.90, 34.6 ± 0.17, and 50.59 ± 0.75 *μ*g/ml, respectively. The Trolox equivalent values of fraction in oxygen radical absorbance capacity (ORAC) assay were 0.34 ± 0.04, and the EC_50_ values in cellular antioxidant activity were 91.24 ± 0.32 *μ*g/ml. The xanthine oxidase inhibition capacity of fraction (100 *μ*g/ml) was 96.4 ± 0.02% *μ*g/ml. The comet assay analysis showed that this fraction (25–100 *μ*g/ml) protects human lymphocytes against H_2_O_2_-induced DNA damages significantly. The IC_50_ values of cytotoxicity assay were 248.145 ± 35.56 and 227.14 ± 16.51 *μ*g/ml after 24 and 48 h, respectively. There has been great attention to the valorization of waste materials. Recovered fraction could be considered as a proper antioxidant, DNA damage-protection agent, and xanthine oxidase inhibitor. Using a nontoxic solid phase such as Amberlite is a fruitful way to concentrate bioactive ingredients which can be used in pharmaceutical and nutraceutical industry.

## 1. Introduction

Steam distillation or hydrodistillation is the most utilizing method to obtain essential oils from aromatic plants [1]. *Rosa damascena* Mill (R. *damascena*) essential oil is well known in the perfume industry, which is a product of the hydrodistillation method.

Every year during the harvest season and production of essential oil, the low yield of essential oil production leads to generate bulky waste [2]. These byproducts are the cause of ecological problems. Consequently, less waste production and the recovery of respected substances with biological activities are considered in this industry [2].

Two types of water and solid waste are present in the hydrodistillation method [2]. Solid waste is reported as a rich source of polyphenols and polysaccharides [2, 3].

Waste water is known as a biopollutant, resistant to decompose, and source of soil pollution; hence, it is necessary to manage this waste [2, 4].

Traditional extraction techniques require long-time processes, high amount of samples, and organic solvents. The drawbacks of these solvents are high cost and poses negative impact on health and environment [5]. Consequently, the development of precise, cost-effective, and environmental friendly method is considerable [6].

Amberlite is the trade name of a series of ion-exchange resins. These resins are known as nonpolar adsorbents which are applied to absorb hydrophobic compounds with molecular weight less than 20,000. Amberlite XAD-7 is moderately polar XAD resin and contains acrylic-ester-resin. Therefore, it possesses intermediate polarity, relatively hydrophilic character, and homogeneous nonionic structure [7].

Polyphenols are the major component of waste waters, and different methods are used to recover several valuable substances in this waste. Quercetin, kaempferol, and ellagic acid have been isolated. The polyphenol-enriched fractions show various biological activities such as tyrosinase inhibition, anti-inflammatory, and antiproliferative effects [2, 4, 8].

In this study, the polyphenol-rich fraction was isolated from *R. damascena* waste waters by Amberlite column as the stationary phase. The phenolic compound content and antioxidant activity of byproduct is compared before and after using the column to realize the efficacy of these types of column to concentrate active compounds. Furthermore, XO inhibition and protective effects against DNA damage induced by H_2_O_2_ on isolated human lymphocytes of the fraction were measured to determine the biological activity of separated fraction.

## 2. Materials and Methods

*R. damascena* Mill were collected from Meymand (Fars Province, Iran) during April 2017. It was authenticated (voucher no. MPPRC-99-1) by Dr. MR. Moein and deposited in the Museum of Medicinal Plants, Department of Pharmacognosy, Shiraz University of Medical Sciences, Shiraz, Iran. Residual water was collected during hydrodistillation of *R. damascena* flowers during April-May 2017 in Meymand (Fars Province, Iran).

### 2.1. Extraction

Liquid byproduct was filtered, freeze-dried by freeze dryer (Zirbus, Germany), and ground into powder. The powder was suspended in water containing trifluoroacetic acid (TFA) (Samchun, Korea) (0.3%) and poured into the decanter, where it was shaken adequately with ethyl acetate (Samchun, Korea). This extraction procedure was repeated three times, and the aqueous phase was collected in each repeat. The aqueous phase was loaded on Amberlite XAD-7 (2.5 × 45 cm) (Sigma-Aldrich, St Louis, MO, USA), and the stationary phase was rinsed with water containing TFA. Then, for the last time, the stationary phase was rinsed with methanol containing TFA, and then the extract was collected and concentrated using a rotary evaporator.

### 2.2. Determination of Total Phenolics, Flavonoids, and Anthocyanin Content

The total phenolic compounds were measured by using Folin–Ciocalteu reagent and different concentrations of gallic acid (Sigma-Aldrich, Co. LLC, USA) as standard. All samples were performed in triplicate.

The total phenolic content was calculated from the calibration curve, and the results were expressed as mg of gallic acid equivalent per g dry weight [9].

Flavonoid content was determined by the colorimetric method. Quercetin (Sigma-Aldrich, Co. LLC, USA) was used as the standard for total flavonoid content measurement. This content was calculated from a calibration curve, and the result was expressed as mg quercetin equivalent per g dry weight of the sample. Data were recorded as mean ± standard deviation (SD) for three replicates [9]. Total anthocyanin was measured according to the pH differential spectroscopic method [10].

### 2.3. DPPH Free Radical Scavenging Assay

In this assay, the antioxidant activity was measured by their reaction with DPPH^•^ (Sigma-Aldrich, Co. LLC, USA) as a free radical and led to discoloration of this molecule.

In brief, DPPH solution was mixed with of sample solutions (12.5–3200 *μ*g/ml) in the 96-well microplate; after incubation (30 min, 25°C), the absorbance was measured at 492 nm [11]. The result was used to determine the concentration of the extracts required to scavenge 50% of the DPPH free radicals (IC_50_), and quercetin (3.12–1600 *μ*g/ml) was used as a standard.

### 2.4. Nitric Oxide Radical Scavenging Assay

In this assay, the mixture of sodium nitroprusside (Sigma-Aldrich, Co. LLC, USA) and a sample was incubated at 27°C for 150 min. Then, Griess reagent was added to the mixture and then incubated at room temperature for 5 min. As a final point, absorbance was measured at 542 nm [11]. Nitric oxide radical scavenging was determined as follows:

(1) $$\begin{array}{c} \left[\frac{\left(A_{1}-A\right)}{A_{1}}\right]\times 100, \end{array}$$

where *A*_1_ denotes control absorbance and *A* denotes test absorbance.

### 2.5. Ferric-Reducing Antioxidant Power (FRAP) Assay

The FRAP reagent contains TPTZ solution (2,4,6-tripyridyl-s-triazine) (10 mM in HCl), FeCl_3_ (20 mM in H_2_O), and acetate buffer (300 mM, pH = 3.6). The fresh mixture was prepared and incubated at 37°C (10 min). Subsequently, 180 *μ*L of FRAP reagent and 20 *μ*L of each sample were mixed. Finally, the absorbance of the reaction mixture was measured at 593 nm after incubation at 37°C for 10 min. The concentration ranges of samples and standard were 12.5–3200 *μ*g/ml. Results were reported as IC_50_ [10].

### 2.6. Oxygen Radical Absorbance Capacity (ORAC) Assay

The procedure was performed by the Polar star omega device (BMG LABTECH GmbH, Germany). The final assay mixture contained fluorescein (Sigma-Aldrich, Co. LLC, USA) (10 nm), AAPH (Sigma-Aldrich, Co. LLC, USA) (240 mM), and sample (25–400 *μ*g/ml) or phosphate buffer as the blank. The decline of mixture fluorescence intensity was recorded every 90 s per cycle. Different concentrations of Trolox (Sigma-Aldrich, Co. LLC, USA) (3–50 *μ*g/ml) were used to give a standard curve to compare the ORAC values of samples. The data were analyzed by data analysis software (MARS). The difference between the “area under the fluorescence decay curve” (AUC) of blank and each sample was expressed as Trolox equivalents (TE) [9].

### 2.7. Xanthine Oxidase (XO) Assay

The inhibition effects of the sample on XO activity were done according to the kit protocol. (Xanthine oxidase activity kit, Sigma-Aldrich). The xanthine oxidase activity of the sample and vitamin C at 100 *μ*g/ml concentration were measured. XO inhibitory activity was evaluated by the following formula:

(2) $$\begin{array}{c} \%\text{ enzyme inhibition}=\left(1-\frac{b}{a}\right)\times 100, \end{array}$$

where “*a*” is the activity of the enzyme without sample and “*b*” is the activity of XO with sample [9].

### 2.8. Antioxidant Assay for Cellular Antioxidant Activity (CAA)

The human liver cancer cell line (HepG2) was obtained from Pasteur Institute (Tehran, Iran). They were maintained at 37°C in an incubator under 5% CO_2_ and cultured in RPMI 1640 supplemented with 10% (v/v) fetal bovine serum (FBS), 100 U/mL penicillin, and 100 mg of streptomycin/ml. After 80% confluency, cells were harvested by trypsin.

HepG2 cells (6 × 10^4^ cells/well) were incubated for 24 h. Then, the medium was removed, and cells were washed with phosphate buffer 3 times. The wells were treated with 2,7-dichlorodihydrofluorescein diacetate (DCFH-DA) (Sigma-Aldrich, Co LLC, USA) and with either quercetin standards or (0.4–2 *μ*g/ml) samples (50–3200 *μ*g/ml). The microplate was incubated (60 min at 37°C). Then, the liquid was removed, and cells were washed by PBS. Finally, free radical initiator solution (AAPH) was added to the wells, and the plate was read on the Polar star omega device [9].

### 2.9. Cytotoxicity Assay

The cell viability assay was determined by a modified 3-(4,5-dimethylthiazol-2-yl)-2,5-diphenyl tetrazolium (MTT) assay. In brief, 10^4^/well HepG2 cells were seeded in 96-well culture plates and allowed to grow 24 h followed by treatment with samples (50–800 *μ*g/ml) for 24 and 48 h. The medium was removed, MTT solution was added, and incubated at 37°C for 4 h. The generated formazan was solubilized with DMSO. The absorption was measured at 570 nm [12].

### 2.10. Comet Assay

#### 2.10.1. Isolation of Human Lymphocytes

Informed consent was obtained from all volunteers, and all procedures were conducted corresponding to the Declaration of Helsinki. Peripheral blood was taken from the volunteers (10 healthy people, 25–30 years old, nonsmokers, and no history of smoking or chronic use of medication). Samples were collected into tubes containing 10% EDTA as an anticoagulant agent. 5 ml of blood was diluted with PBS, and the suspension was placed over the lymphocyte separation medium carefully and then centrifuged; gradient-separated lymphocytes were recovered, diluted with PBS, and centrifuged again. The pellets were resuspended in PBS, and then cells were counted in a Neubauer chamber. Cell viability was checked by trypan blue dye exclusion technique, and cell concentration was adjusted to 5000 cells/ml [9].

#### 2.10.2. Alkaline Comet Assay

The alkaline comet assay was performed according to the guidelines of Singh et al. with some modifications [13]. The cells were treated with different concentrations of sample and H_2_O_2_ (100 *μ*M) simultaneously (20 min in the dark at 4°C) to inhibit DNA repair followed by the induced oxidative DNA damage. H_2_O_2_ solution in PBS was used as a positive control. The cells were harvested and centrifuged at 3000 rpm for 10 min and then washed with PBS. The cell pellets were mixed with 100 *μ*l of low melting point agarose (0.75% w/v), then spread on a microscopic slide precoated with normal melting agarose (1%), covered with a coverslip, and kept for 10 min at 4°C. After removing the coverslips, the slides were immersed in freshly prepared cold lysing solution (2.5 M NaCl, 100 mM Na_2_EDTA, 10 mM tris, 1% (v/v) triton X-100, 10% DMSO, pH 10.0) at 4°C for at least 2 h. The slides were presoaked in freshly prepared alkaline electrophoresis buffer for 30 min. Electrophoresis was carried out (45 min at 4°C). All procedural steps were performed under yellow light conditions to minimize additional DNA damage. Then, slides were placed vertically in a neutralizing tank and washed with a neutralizing solution (0.4 m tris HCl buffer, pH 7.5). Lastly, the slides were stained with propidium iodide (Sigma-Aldrich, USA) (20 *μ*g/ml) dispensed directly onto the slides and covered with a coverslip. The slides were studied by a fluorescent microscope (Olympus-BX61). All experiments were performed at least three times. For each slide, 50 selected cells were analyzed with CometScore software.

#### 2.10.3. Statistical Analysis

The results were analyzed using one-way analysis of variance (ANOVA), followed by Tukey's post hoc tests done by SPSS (version 20). Values were expressed as mean ± SEM. *p* value of less than 0.05 was considered significant.

## 3. Results and Discussion

### 3.1. Phenolic Content

*R. damascena* (Damask rose) is considered as a significant industrial member of the Rosaceae family, and rose oil is a product of hydrodistillation of this aromatic plant [1]. Waste water and solid waste biomass have been known as byproduct of this hydrodistillation process, and they have been noted as sources of potent active compounds [2].

In this study, we used Amberlite to improve the content of phenolic compounds in separating fractions. Our results confirm the efficacy of this type of column to achieve enrich fraction from waste water. For instance, our results showed phenolic content (phenol, flavonoid, and anthocyanin) of the fraction (after loading on column) was amplified considerably compared with waste water (Table 1). Using the XAD-7 Amberlite in the extraction process as the stationary phase would be able to separate nonpolar compounds from the extract and increase the concentrations of phenolic compounds. These results are consistent with the data of the previous study, which explains that this type of column increases the phenolic content of *Berberis integerrima* fraction. It can be related to the capacity of this column to interact with nonpolar/moderate polar phenolic compounds with intense hydrogen bonds between the ester groups on the column surfaces and hydroxyl groups of these compounds.

Indeed, this column can reduce protein and polysaccharide content of the sample and lead to increase in phenolic concentration content [9].

Recently, there is great attention on the valorization of crude plant materials for developing natural products. Due to the toxicity of organic solvents, using nontoxic solid phase such as Amberlite is a fruitful way for the concentration of bioactive ingredients. Meanwhile, it is important to design one or two steps of fractionation process to enrich these components.

Aroma compounds, polyphenols, and polysaccharides were recovered compounds in residual mass [2] such as kaempferol and quercetin [14, 15].

Schieber et al. in their study reported kaempferol 3-O-glucoside as the most predominant substance in distilled rose petals. This sample was rich in kaempferol glycosides and kaempferol aglycone as well [16].

The results of phenol and flavonoid contents in our study were significantly more than those which were reported by Abdel-Hameed et al. These values in water byproducts were 48.27 ± 1.27 and 16.68 ± 0.73 mg gallic acid equivalent/g extract for phenol and flavonoid, respectively. ESI (−ve): MS analysis of this byproduct supports the presence of phenolic compounds belonging to hydrolysable tannins and flavonoids [17].

### 3.2. Antioxidant Assay

The radical quenching activities of the samples were measured by using various methods (Table 2). The radical scavenging ability of the fraction increased considerably in comparison with the waste in ABTS, DPPH, and nitric oxide assays. Furthermore, the fraction was more powerful to reduce Fe^3+^ to Fe^2+^ in the FRAP method in comparison with waste water.

These noticeable antioxidant activities of a fraction are related to increasing the amount of phenolic compounds after loading on the column. Besides phenolic compounds, antioxidant capacity can be due to the presence of residue of essential oils and other nonvolatile compounds in the rose biomass and waste waters [18–20].

The previous study represents the rose water byproduct as a potent free radical scavenging agent in different methods such as DPPH radical scavenging, total antioxidant capacity, and reducing power activity [17].

From other antioxidant assays (ORAC), the ability of fraction to hydrogen donation was measured [9]. The Trolox equivalent values for quercetin and the fraction in the ORAC analysis were 0.72 ± 0.021 and 0.34 ± 0.04, respectively.

In the CAA assay, the antioxidant activity of the sample in the cells was predictable. The EC_50_ values for cellular antioxidant activity were 55.51 ± 0.11 and 91.24 ± 0.32 *μ*g/ml for quercetin and the fraction, respectively. On the other hand, the fraction could inhibit oxidation of 2′,7′-dichlorofluorescein (DCFH) to DCF by peroxyl radicals in the cells [9].

The analysis of the xanthine oxidase inhibition capacity of the fraction showed that 50 *μ*g/ml concentrations of the fraction and vitamin C inhibit this enzyme by 96.4 ± 0.02% and 92.8 ± 0.05%, respectively.

This powerful activity would be related to high phenolic compounds of this fraction after loading on the column. These compounds were introduced as potent XO inhibitors. There have not been reported XO inhibition activities of this fraction yet. Wedler et al. showed that polyphenol-enriched fraction was a potent tyrosinase inhibitor. As this enzyme has a role in oxidative stress and oxidative damages, new XO inhibitors, particularly natural inhibitors, are really considerable in recent research studies [9].

Anti-inflammatory [4] and antiproliferative effects in immortalized human keratinocytes are other reported biological activities of this fraction [8].

### 3.3. Cytotoxicity

In cytotoxicity assay, HepG2 cells were exposing to different concentrations of fraction for 24 and 48 h. There is significant toxicity at a concentration of 100 *μ*g/ml and higher (*p* < 0.001) in this cell line. The IC_50_ of fraction for 24 h and 48 h incubation were 248.145 ± 35.56 and 227.14 ± 16.51 *μ*g/ml, respectively. Wedler et al. reported that rose oil distillation waste water had dose-dependent antiproliferative activity on HaCaT cells. The reported IC_50_ was 9.78 *μ*g/ml [8].

### 3.4. Comet Assay Analysis

Reactive oxygen species (ROS) are generated in intracellular or extracellular pathways. Excessive ROS contribute to cellular damages. Among different damages, DNA oxidative damages play a serious role in aging, cancer, and other diseases [21]. In this study, the probable protective effect of fraction on H_2_O_2_-induced DNA damages in human leukocytes was measured by comet assay analysis.

**Figure 1:**
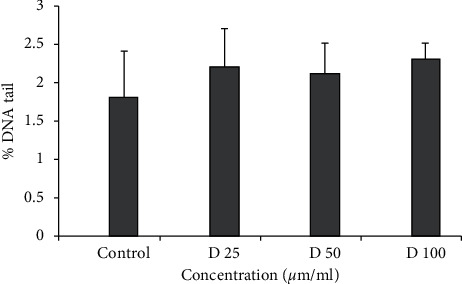
DNA damage of human lymphocytes treated with fraction. Human lymphocytes were incubated for 15 min at 4°C with different concentrations of fractions (25, 50, and 100 *μ*g/ml).

**Figure 2:**
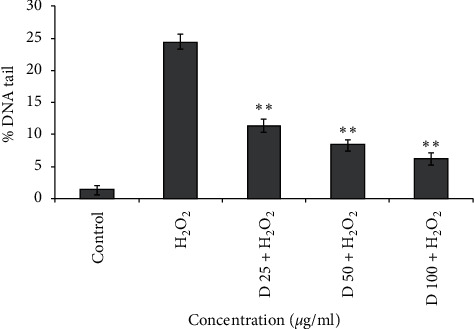
Effect of fraction on lymphocyte DNA damage induced by 50 *μ*M H_2_O_2_. Human lymphocytes were incubated for 15 min at 4°C with a combination of 50 *μ*M H_2_O_2_ with different concentrations of fractions (25, 50, and 100 *μ*g/ml).

In this assay, cells were exposed to H_2_O_2_ as a source of ROS and sample as an antioxidant simultaneously, for tiny time. Generally, more antioxidant activity leads to more reduction in DNA breaks [22]. For the fraction, a range of concentrations (25–100 *μ*g/ml) did not show any genotoxicity and could be considered as a nongenotoxic concentration (Figure 1). The cells exposed to H_2_O_2_ solely showed significant DNA strand breaks, while lymphocyte treated with 25, 50 and 100 *μ*g/ml fraction and H_2_O_2_ simultaneously showed a significant reduction of DNA damage level (*p* < 0.01) (Figure 2). These results are in favor of antioxidant activity as well as a high amount of phenol, flavonoid, and anthocyanin of this fraction.

It seems that the phenolic compounds reduce free radicals' side effects before they induce any DNA damages. According to phenolic compounds structure, the hydrogen atom of them can remove hydroxyl radicals generated by H_2_O_2_ [22, 23]. Moreover, the interaction between the dihydroxy group in phenolic structure and the transition metal such as iron and copper can inhibits the Fenton reaction [9].

Nowadays, there is a great demand on consumption of antioxidant as chemopreventive agents, so isolation and detection of potent antioxidant is the first step to produce new supplements. So, in the next step, it is necessary to develop and apply chemical techniques to characterize the chemical structure of compounds which are responsible of these biological effects. This step is our research limitation, but it can be a really important approach for valorization of rose waste since this characterization can reveal the potential of rose wastes which is still unexploited.

## 4. Conclusion

This study suggested that Amberlite, as a stationary phase, can be served to expand the phenolic content in the waste fraction instead of expensive and toxic solvents.

This fraction can be considered as an antioxidant, xanthine oxidase inhibitor, and DNA damage-protection agent, which could be attributable to the high amount of phenolic compound. The byproduct of industrial rose oil production can be considered as a proper source of bioactive compounds, and extraction of these substances in further investigations led to the recognition new products and waste valorization.

## Figures and Tables

**Table 1:** Total phenolic, total flavonoids, and total anthocyanin content of waste water before and after (phenolic rich fraction) loading on the column.

Sample	Total flavonoids (mg QE/1 g extract)	Total phenolic (mg GAE/1 g) extract	Total anthocyanins (mg/g^−1^)
Waste water	29.90 ± 0.13	167.76 ± 0.29	0.31 ± 0.004
Phenolic rich fraction	186.75 ± 0.27	381.45 ± 0.41	3.5 ± 0.31

**Table 2:** Antioxidant activities of the extract of waste water before and after (phenolic rich fraction) loading on column.

Sample	DPPH (IC_50_, *μ*g/mL)	Nitric oxide scavenging ability % (200 *μ*g/mL)	FRAP (IC_50_, *μ*g/mL)	ABTS (IC_50_, *μ*g/mL)>
Waste water	226.66 ± 1.25	38.09 ± 0.02	126.03 ± 0.11	241.43 ± 0.33
Phenolic rich fraction	63.21 ± 0.90	60.36 ± 0.05	34.6 ± 0.17	50.59 ± 0.75
Quercetin	26.51 ± 0.06	—	8.69 ± 0.03	25.64 ± 0.02

## Data Availability

The data used to support the findings of this study are available from the corresponding author upon request.
